# Respiratory symptoms and lung function in relation to wood dust and monoterpene exposure in the wood pellet industry

**DOI:** 10.1080/03009734.2017.1285836

**Published:** 2017-03-01

**Authors:** Håkan Löfstedt, Katja Hagström, Ing-Liss Bryngelsson, Mats Holmström, Anna Rask-Andersen

**Affiliations:** a Department of Occupational and Environmental Medicine, Faculty of Medicine and Health Örebro University, Örebro, Sweden;; b Division of Ear, Nose and Throat Diseases, Department of Clinical Science, Intervention and Technology, Karolinska Institutet, Stockholm, Sweden;; c Department of Medical Sciences, Occupational and Environmental Medicine, Uppsala University, Uppsala, Sweden

**Keywords:** Asthma, lung function, occupational exposure, respiratory symptoms, rhinitis, wood dust

## Abstract

**Introduction:**

Wood pellets are used as a source of renewable energy for heating purposes. Common exposures are wood dust and monoterpenes, which are known to be hazardous for the airways. The purpose of this study was to study the effect of occupational exposure on respiratory health in wood pellet workers.

**Materials and methods:**

Thirty-nine men working with wood pellet production at six plants were investigated with a questionnaire, medical examination, allergy screening, spirometry, and nasal peak expiratory flow (nasal PEF). Exposure to wood dust and monoterpenes was measured.

**Results:**

The wood pellet workers reported a higher frequency of nasal symptoms, dry cough, and asthma medication compared to controls from the general population. There were no differences in nasal PEF between work and leisure time. A lower lung function than expected (vital capacity [VC], 95%; forced vital capacity in 1 second [FEV_1_], 96% of predicted) was noted, but no changes were noted during shifts. There was no correlation between lung function and years working in pellet production. Personal measurements of wood dust at work showed high concentrations (0.16–19 mg/m^3^), and exposure peaks when performing certain work tasks. Levels of monoterpenes were low (0.64–28 mg/m^3^). There was no association between exposure and acute lung function effects.

**Conclusions:**

In this study of wood pellet workers, high levels of wood dust were observed, and that may have influenced the airways negatively as the study group reported upper airway symptoms and dry cough more frequently than expected. The wood pellet workers had both a lower VC and FEV_1_ than expected. No cross-shift changes were found.

## Introduction

Current environmental and energy objectives in Sweden are to replace fossil fuels with renewable sources of energy, such as wood pellets, in order to decrease carbon dioxide emissions. The production of wood pellets started in Sweden in the 1980s and has increased significantly recently. In 2015, the pellet production was 1.67 tons coming from almost 65 plants, corresponding to an energy production of 8 TWh. The raw material consists of wood by-products from the sawmill industry, particularly shavings and sawdust, mainly from soft woods such as spruce (*Picea abies*) and pine (*Pinus sylvestris*).

Exposures to wood dust and monoterpenes are commonly measured in the wood-refining industries. Personal wood dust exposure levels at wood pellet production plants have been noted to be between <0.60 and 8.0 mg/m^3^ as inhalable dust and levels of monoterpene (only α-pinene) were <0.28 to 25 mg/m^3^ (1). Standard methods for measuring dust describe average levels of dust in the air during a shift, but no peaks in exposure during the working day. Peaks in exposure can, however, be measured using personal real-time monitors (2–4).

Exposure to wood dust may cause symptoms in the skin, eyes, nose, and airways. A literature review concluded that wood dust exposure is a risk factor for the development of asthma, chronic bronchitis, rhino-conjunctivitis, and chronic impairment of lung function (5). Eye irritation and upper airway symptoms have been reported after exposure to spruce and pine dust levels in the range 0.1–6.3 mg/m^3^ (6–9). Objective impairment of nasal function has also been reported in workers exposed to wood dust: impaired nasal peak expiratory flow (nasal PEF) (10), impaired mucociliar clearance, and rhinomanometry (11). There are also indications of reduced lung function after long-term exposure to dust from soft wood at levels of 1.3 mg/m^3^ (12) and 3.8 mg/m^3^ (13). There was a low pre-shift lung function among workers in a joinery shop (14), and in a study of woodworkers in furniture industry an association was found between average dust exposure and cross-shift forced vital capacity in 1 second (FEV_1_) (9). Increased bronchial responsiveness has been reported in workers sawing pine (6,15). The International Agency for Research on Cancer (IARC) has classified wood dust as carcinogenic, particularly for cancers of the nasal cavities and paranasal sinuses, mainly as a result of exposure to hardwoods, but there may also be a much smaller excess risk of sinonasal cancer associated with softwoods (16–18).

Monoterpenes are irritating to skin, eyes, and mucous membranes, and can cause both non-allergic and allergic contact dermatitis (1,19), and consequently woodworkers have a higher risk of developing hand eczema (20). The most abundant monoterpenes in softwood are α-pinene, β-pinene, and Δ^3^-carene (21). They can be taken up through the lungs, the gastro-intestinal tract, and intact skin (19,22). Results from animal studies have suggested that high concentrations of Δ^3^-carene might lead to asthma (23), and that skin sensitization can increase lung reactivity (24). Due to emissions during storage of wood pellets, there is a risk for carbon monoxide (CO) intoxication both in large-scale bulk transportation (25) and in smaller store-rooms in households. There are several reports of life-threatening concentrations of CO being produced by wood pellets and other wood products (26).

To our knowledge, there is no systematic survey of health status related to airway exposure among employees at wood pellet plants. Therefore, the present investigation was performed to study the health status, mainly in the upper and lower airways, among workers at six pellet-producing plants and the relationship to exposure to wood dust and monoterpenes.

## Material and methods

### Industries

Six plants in central Sweden involved in industrial production of wood pellets were included in the study. At all plants, the production process was similar, and at two of them briquettes were also produced. Shavings and sawdust, stored outside or under roof cover, were used as raw material. Shavings could be used immediately for pelleting, but sawdust had to be dried before the process, which started with grinding the sawdust and shavings before pressing at a temperature of around 100 °C. After cooling, the pellets were stored. The sawdust used in the industries consisted of equal amounts of spruce and pine except for one plant, where oak and beech were also used. There was a rapid turnover of raw material, and mold problems were not reported from any of the production plants.

### Study population

All employees involved in production at the six wood pellets plants (39 men) were invited to participate in the study, and all accepted. The study was approved by the Ethics Committee of the Örebro County Council (1012/00).

### Questionnaire

A questionnaire was mailed to the participants with questions about work, leisure time, dwelling, smoking habits, exposure to air pollutants, and symptoms from the respiratory system. The answers to the questionnaires were compared with answers to the same questions from controls from the general population. These controls came from other studies on respiratory symptoms and asthma carried out in Sweden during the same time period (8,27,28).

### Clinical tests

Subjects were offered a medical examination performed at the workplace. Lung function was examined with pre- and post-shift spirometry and registered with a vitalograph (Vitalograph 2150) according to guidelines established by the American Thoracic Society (ATS) (29). Swedish reference values, which take gender, age, and height into account, were used to calculate predicted lung function values (30). The results of the pre- and post-shift spirometry were compared with the lung function of 118 male foundry workers without airway exposure (31).

Maximal expiratory airflow through the nasal cavities was investigated with nasal PEF (32). The participants used a Mini-Wright standard PEF-meter with an anesthetic mask connected. Three to four registrations were performed in an upright position and after a maximal inhalation. The highest value was used in the analysis, at four self-chosen occasions daily during one week. Each person served as his own control, and the results were thus compared between work and leisure time for each individual.

Blood was taken from each subject for analysis of concentrations of IgE antibodies to a mixture of common airborne allergens (Phadiatop®, Phadia AB, Uppsala, Sweden). Serum samples were analyzed at the Department of Microbiology, Örebro University Hospital. One sample was analyzed at the Chemical Laboratory, Central Hospital, Västerås. IgE concentrations ≥35 kU/L were regarded as positive (33).

### Exposure assessment

Personal exposure to wood dust and monoterpenes was measured for 24 of the participants. The measurements ran in parallel with the lung function tests, i.e. the same day as the cross-shift spirometry. The workers worked morning and afternoon shifts as well as day shifts. The exposure measurements have been described and presented in detail elsewhere (34).

Briefly, exposure was measured with pumped sampling of wood dust as ‘total’ dust (25 mm cellulose acetate filter in an open-faced, antistatic cassette with an airflow of 2 L/min). The filters were gravimetrically determined (LOD 0.001 mg) after being conditioned (48 h at 20 ± 1 °C and at a relative humidity of 50% ± 3%) before and after the sampling. Monoterpenes, α-pinene, β-pinene, and Δ^3^-carene, were collected by diffusive sampling. Both samplers were placed in the worker’s breathing zone.

Dust concentration for 18 of the participants was also monitored continuously using a personal data-logging real-time aerosol monitor (DataRAM; MIE, Inc., Bedford, MA). The DataRAM was placed on the belt of the worker, and the dust concentration was measured every twentieth second. A peak was defined as successive registrations over a threshold of 0.4 mg/m^3^ (20% of the Swedish occupational exposure limit [OEL]). Three variables were created to examine if the number of peaks, the time over a threshold value, or the amount over a threshold value affected health.

### Dose–response relationships

Chronic effects were monitored using vital capacity (VC) and FEV_1_ in the morning as well as answers in the questionnaires concerning time at the workplace and time with current work tasks. Acute effects during the day were monitored by following changes in VC and FEV_1_ in relation to the mean dust level, the number of peaks/h, the time over the threshold value/h, and the level over the threshold value/h.

### Statistical methods

Arithmetic mean, standard deviation (SD), and range are shown for quantitative data; descriptive statistics and geometric means are also presented for exposure measurements. Differences between study population and the control group were analyzed using Student’s *t* test. Differences in qualitative variables between study population and reference data were analyzed using a chi-square test. Possible relationships between exposure and health outcome were studied using regression analysis. Results were considered to be statistically significant at *p* < 0.05. All analyses were performed in SPSS for Windows v. 12.0.1.

## Results

### Questionnaire

The mean age of the 39 men in the study group was 38 years (range 21–63 years) (Table 1). A majority of them were never-smokers (69%), and only 10% were smokers.

**Table 1.** Distribution of background variables among study subjects (*n* = 39).

	Number	%
Age (years)		
<30	7	18
30–39	20	51
≥40	12	31
Smoking habits		
Smokers	4	10
Non-smokers	27	69
Ex-smokers	8	21
Time with present working tasks (years)		
<5	16	41
5–9	21	54
≥10	2	5
Time at present workplace (years)		
<4	15	38
5–9	20	51
≥10	4	10
Part-time farming		
Yes	10	26
No	29	74
Part-time forestry		
Yes	11	28
No	28	72

The subjects in the study group had been employed at their current plant for an average of 7 years (0–44 years) and had been working with their current work task for an average of 5 years (0–17 years). A majority (72%) of the workers used bio fuels, mainly wood pellets (51%), for heating of their homes. More than one-quarter worked part time with farming and/or forestry (Table 1).

Earlier or current nasal symptoms without contemporaneous infection were reported by 46%, and half of these reported nasal symptoms mainly during work (Table 2). The most common symptoms, which also occurred more frequently among exposed than controls, were nasal blockage, sneezing, and dryness. Among the exposed workers, 26% reported sneezing, and 28% reported blockage.

**Table 2.** Prevalence of symptoms from the upper and lower airways among study subjects (*n* = 39) compared with controls (results shown in percent).

	Exposed	Controls
Nasal symptoms:		
Sneezing	26^a^	11^b^
Irritation	8	7^b^
Blockage	28^a^	9^b^
Dripping	10	8^b^
Mucous secretion	8	–
Dryness	20^a^	6^b^
Impaired sense of smell	8	4^b^
Lower airway symptoms:		
Asthma attack last year	8	3^c^
Doctor-diagnosed asthma	15^a^	7^d^
Asthma medication	13^a^	5^c^
Nightly breathlessness	8	6^c^
Work-related febrile attacks	0	3^e^
Dry cough	41^a^	15^e^
≥3 months per year	18^a^	5^e^
Cough with phlegm	26	22^d^
≥3 months per year	5	9^d^

a *p* < 0.05.

b Åhman et al., 1995, *n* = 112 (8).

c Björnsson et al., 1998, *n* = 6031 (27).

d Lembke et al., 2004, *n* = 900 (28).

e Rask-Andersen and Lemke unpublished data (Swedish report).

The workers in the wood pellet production plants reported more nasal symptoms, asthma attacks, nightly breathlessness during the last year, asthma medication, and cough, but a statistically significant difference was only noticed for nasal symptoms, dry cough, doctor-diagnosed asthma, and asthma medication (Table 2). Five of six subjects with physician-diagnosed asthma reported at the medical examination that they had asthma before they had started working in the production of wood pellets. The sixth subject developed asthma during employment at the wood pellet plant and had a family history of asthma. Two of the asthmatics and another participant reported attacks of asthma and awakenings with dyspnea during the last year, the latter in connection with pneumonia. Dry cough (41%) and sneezing (26%) were the most common symptoms in the study group. Dry cough was more common than among the controls. None in the study group reported work-related febrile attacks.

### Clinical tests

Spirometry before shifts was lower when compared to reference data among both exposed and controls, measured as VC (94.9%, *p* < 0.01 versus 94.2%, *p* < 0.001), but FEV_1_ was decreased in the controls only (96.4%, *p* = 0.074 versus 96.7%, *p* < 0.05, Table 3). However, the changes in VC and FEV_1_ over a shift were small (VC −0.2% versus −0.6%; FEV_1_ +0.1% versus +0.2%). There was no statistically significant difference in lung function between exposed and controls. The subjects with physician-diagnosed asthma had lower VC (*p* = 0.052) and FEV_1_ (*p* < 0.01) compared to the other participants. One study subject was excluded from the analysis of lung function due to defectively performed spirometry.

**Table 3.** Pulmonary function (VC, FEV_1_, and FEV%) in exposed subjects (*n* = 38) and foundry worker controls (*n* = 118) before and after shift.

	Exposed *n* = 38			Controls *n* = 118		
	Mean (% of predicted)	SD	Range	Mean (% of predicted)	SD	Range
FEV_1_						
before	96.4	13.3	62–129	96.7	11.5	62–128
after	96.5	12.8	59–129	96.9	11.6	63–128
difference	+0.1	3.1	−7–7	+0.2	4.0	−11–11
VC						
before	94.9	11.7	67–117	94.2	10.8	72–125
after	94.6	10.1	70–114	93.6	10.5	73–122
difference	−0.2	3.2	−7–6	−0.6	3.1	−8–16
FEV%						
before	101.9	8.9	68–121	102.9	8.1	66–118
after	102.2	8.9	64–114	103.7	7.8	66–117
difference	+0.2	2.9	−8–6	+0.8	4.0	−17–11

FEV%: FEV_1_/VC; FEV_1_: forced vital capacity in 1 second; VC: vital capacity.

There was no correlation observed between lung function and number of years with current working tasks. However, when the subjects were divided into two groups (0–4 years versus ≥5 years with current working tasks), the group with longer exposure tended to have a lower lung function, but the difference did not attain statistical significance (Table 4). Neither personal measurements (wood dust and monoterpenes) nor peak exposures measured with DataRAM showed any correlation with acute effects on lung function. At one of the plants, a decrease of VC (*p* < 0.05) over a shift was observed.

**Table 4.** Pulmonary function (VC, FEV_1_, and FEV%) among study subjects (*n* = 38) before shift in relation to years of employment in current working tasks.

	*n*	FEV_1_	VC	FEV%
Current working tasks <5 years	16	98	98	100
Current working tasks ≥5 years	22	95	93	103

FEV%: FEV_1_/VC; FEV_1_: forced vital capacity in 1 second; VC: vital capacity.

Nasal PEF measurement did not show any differences between work and leisure time.

### Exposure assessment

The study subjects worked morning and afternoon shifts as well as office hour shifts. During the morning and afternoon shift work, operations included: loading of raw material, monitoring from the control room, maintenance work, repairs, and sweeping and cleaning of truck engines with compressed air. Day shift workers did, to a larger extent, drive trucks and work with bagging (34).

Almost all of them reported exposure to dust at work, but only 13% reported dust exposure during leisure time. Contact with other air pollutants at various levels was also reported. Furthermore, almost everybody reported dust exposure during cleaning. The most common cleaning methods were sweeping and cleaning with compressed air and, to a lower extent, vacuum cleaning.

The personal exposure to ‘total’ wood dust varied in the range 0.16–19 mg/m^3^ (geometric mean [GM] 1.7 mg/m^3^), and to monoterpenes, 0.64–28 mg/m^3^ (GM 5.0 mg/m^3^) (Table 5). Eleven of the 24 measurements exceeded the Swedish occupational exposure limit (OEL) for wood dust at 2 mg/m^3^ as ‘total’ dust at the time of the measurements (ASS 2001). All monoterpene levels were far below the Swedish OEL for monoterpenes of 150 mg/m^3^ (ASS 2005). The measurements with DataRAM showed a great variation between employees both within and between industries. Peak dust levels were observed during work tasks including monitoring in the control room, maintenance work, sacking, loading raw material, and sweeping and cleaning with compressed air. The number of peaks (>0.4 mg/m^3^) recorded for each worker varied between 4 and 49 over an 8-h working day. For more details, see Edman and co-workers (34). Analyses of the dose–response relationships did not reveal any statistical significant relationships.

**Table 5.** Stationary and personal exposure levels of wood dust in the wood pellet industry.

		Wood dust (mg/m^3^)		
Measurements	Number	GM	AM	Range
Stationary	49	0.56	2.9	<0.10–34
Personal	24	1.7	3.5	0.16–19

AM: arithmetic mean; GM: geometric mean.

At the date of the study the OEL for wood dust was set for total dust in Sweden. It is, however, better to measure the inhalable fraction since wood dust particle is mainly composed of particles with a median aerodynamic diameter >10 μm (35). Calibration studies have shown that inhalable wood dust concentrations are on average 1.6 to 4 times higher than total wood dust concentrations (1,35–38). The new OEL in Sweden for wood dust is today set to 2 mg/m^3^, indicating that even more of the measurements would have been above today’s OEL if inhalable fraction had been measured.

## Discussion

In this study of 39 workers in six wood pellet-producing plants, there was a significantly higher prevalence of nasal symptoms such as nasal blockage, sneezing, and dryness in the nose. The workers also reported more asthma attacks, more nightly breathlessness during last year, more asthma medication, and more cough than the controls, but only the prevalence of doctor-diagnosed asthma, asthma medication and dry cough was significantly higher than in the controls. One worker, who had asthma in the family, had developed asthma after starting work in pellet production. A slightly lower lung function than expected was also found in the workers producing wood pellets. No acute changes in lung function over shifts were recorded and no effect of increasing time of exposure. The exposure measurements indicated high levels of wood dust, but the levels of monoterpenes were low.

A lower lung function than expected has also been reported in previous studies with similar exposure (12). In this study, the lower lung function before starting a shift could indicate an effect from the accumulated dose of wood dust, but the weak association between lung function and years of exposure as well as the lack of difference compared with the control group did not support such an effect. However, this is a young industry with only two plants that had produced wood pellets for more than 9 years. The mean employment time was 5 years, and only 10% had worked at least 10 years with the present working tasks. Therefore, the exposure time may be too short to induce long-term effects on the lung function. The decreased lung function observed in this study may be an effect of occupational exposures in previous employments (39,40). Maybe the workers had worked in another wood-processing industry before, or they had performed wood-exposing tasks in their spare time.

There was no effect on lung function during a shift. This may be explained by the absence of acute effects on lung function during industrial production of wood pellets, diurnal variation, or an effect that is masked because subjects improved their results, with more experience of spirometry when tested a second time (41).

The high prevalence of airway symptoms is in accordance with results from previous studies in similar environments (6). Five of six asthmatics had their diagnosis before involvement in the production of wood pellets. The raw material is usually kept dry and is turned over relatively quickly, which decreases the risk of mold growth. Mold-contaminated raw material was not found during the visits at the plants, and nobody reported febrile attacks, which may appear at high exposure to molds—so-called inhalation fever (42,43).

Nasal PEF measurements did not show any variation between work and leisure. Once again it is unclear if this depends on an exposure without any effect on nasal airflow, or a real effect hidden by the study design. The common difficulties in registering nasal PEF, variations in time point for registration, and in relation to work and leisure time are some factors that may have decreased the precision in this investigation. There is unfortunately no objective method to measure nasal breathing that has a high correlation to subjective symptoms. In another study, however, daily serial nasal PEF in workers exposed to wood dust correlated to exposure over an observation week (10).

Phadiatop reveals information about sensitization against the most common airborne allergens and disposition to develop allergy (atopy). The proportion of subjects with positive Phadiatop results in the study group did not differ from results in other studies (44–46), which may indicate that the working environment neither leads to increased exposure to the allergens in the test nor increases the risk for development of allergy. However, an increased risk for allergy may be hidden by the fact that allergic persons could have quit their jobs due to difficulties in staying in the dusty environment. This seemed not to be the case, since the turnover of employees at the industries in this study was regarded as low.

The levels of wood dust varied, but were in general high compared to the OEL, with 45% of the measurements over the Swedish OEL. The fact that there were no dose–response relationships in this study might be explained by a few factors. Firstly, the study group was very small, making it hard to detect significant effects. Secondly, the industry was relatively new when the study was performed. But since levels are high compared with other wood-working industries, it is possible that health effects (1) can occur after periods of longer exposure. It is therefore important to continue monitoring the industry. Thirdly, it has been observed in other studies in wood pellet production plants that there is a higher within-worker variation than between-worker variation, which leads to a very high attenuation bias in the material (47). A high attenuation bias can mask even a true dose–response relationship due to an underestimation of the regression coefficient (48–50).

Relatively low levels of monoterpenes were measured in comparison with the Swedish OEL (150 mg/m^3^) and results from previous studies in sawmills and joineries (14,51–54). This indicates that exposure to monoterpenes are of low concern at the production of wood pellets compared to the exposure to wood dust.

The results of this study imply that exposure to monoterpenes was neither a medical nor an occupational hygiene problem during industrial production of wood pellets. However, high levels of wood dust were observed and may have influenced the airways negatively. The study group reported respiratory symptoms more frequently than expected, but there was no obvious effect on lung function based on the methods used. Measures should be implemented to reduce exposure to wood dust in the workplaces to decrease the burden of the airways. In conclusion, this study from the wood pellet industry shows an exposure to wood dust and there was a high prevalence of nose symptoms but no effect on lung function.
